# Practical Notes and Formulæ

**Published:** 1884-03-20

**Authors:** 


### PRACTICAL NOTES AND FORMULÆ.

**A Reliable Tænifuge.**—

℞ Extracti filicis maris ........................... ʒ iss,

Pulveris kamalæ ........................... ʒ ij,

Mucilaginis acaciæ, } ........................ aa ʒ ij,

Syrupi simplicis, }

Aquæ cinnamomi ........................... ad ℥ iij.

M. S. Half to be taken at bed time, and the other half early in the morning.

Mr. J. B. Lawson reports good results from this in the Glasgow Medical Journal, January, 1884.

**Dr. Burrows** says: Oleic acid is good to put on the surface to stop itching, and when bismuth is mixed with it, 1 to 2 drachms to the ounce, it is the best thing I have for scalds and burns up to the third degree of severeness. This also allays the itching of erythema, and is good for the chafed surface of infants.—*Ex.*

**For Dyspepsia with Constipation and Piles.**—

℞ Tinct. nucis vom ........................... ℥ j,
Podophyllin ........................... gr. j.

Triturate thoroughly to dissolve.

Sig. Five drops to be taken in water before each meal.

Appetite will improve and stools become natural in a few days. —*Summary.*

**Iodide of Ethyl.**—Prof. Bartholow says: iodide of ethyl is a very valuable anti-spasmodic, singularly and immediately beneficial in spasmodic asthma, also lessening liability to subsequent attacks. In capillary bronchitis it is conspicuously beneficial, as also in catarrhal pneumonia. In chronic bronchitis it is a most valuable agent, from its local action. It will probably take the place of iodine vapor for respiratory diseases. The dose is gtt. v–xx three or four times a day, by inhalation, generally from a handkerchief.—*Coll. and Clin. Record.*

**Malarial Poisoning** takes the form of fever and ague, or dysentery. In fever and ague recurring in a person who has been subject to it, it is well to give

℞ Quin sulph ........................... gr. iv,
Tinct. digital ........................... m. x,
Ac. phosph. dil ........................... m. xv,
Aq ........................... ℥ j.
Ter in die, with a Pil. Cal. Col. Co., at night, and mercurial tonic in the morning. The poison lying chiefly in the portal circulation, evacuants are indicated, as well as the malarial specific, quinine.

In malarial dysentery, not the putrescent dysentery of camps, it is well to give ipecacuan in full doses. Say you give a drachm of laudanum to a patient, and in two hours, when the stomach is under the influence of the narcotic, pulv. ipecacuan., ʒ i. The patient, for a few hours, will be deadly sick, then vomit, and next day there is a normal stool containing bile, and the dysentery is relieved. If not thoroughly successful in the first attack, repeat the manœuvre.—*Fothergill.*

**Treatment of Chapped Hands and Frosted Feet.**—At a meeting of the Philadelphia County Medical Society, held November 21, 1883, Dr. Carl Seiler called attention to the value of tincture of bensoin in the treatment of chapped hands and frosted feet. He had used it in a number of cases with much success. It is applied by simply painting it on the skin. The stockings may be prevented from sticking to the feet by rubbing some oil over the benzoin.—*Summary.*

**Flatulent Dyspepsia.**—

℞ Potass. chlor.................................ʒ j,

Sodæ bicarb.................................ʒ iiss,

Rhei pulv.................................ʒ ss,

Capsici pulv.................................grs. iv,

Ol. sassafras.................................gtt. ij.

M. Sig. Dissolve in half pint of water, and give tablespoonful immediately after each meal.—*Medical Brief.*

**Local Anæsthesia.**—According to the Medical News, local anæsthesia may be readily produced by applying with a camel's hair brush the following mixture:

℞ Chloral, Camphor, } .................................aa ʒ j,

Morph. sulphat.................................ʒ ss,

Chloroform.................................℥ j.

M. Sig. To be applied with a brush to the area to be incised. —*Med. Summary.*

**Dr. Goodell's Mixture of the four Chlorides.**—The following is known as Dr. Goodell's mixture of the "four chlorides," which he prescribes as an alterative tonic:

℞ Hydrarg. bichlor.................................grs. j-ij,

Liq. arsen. chlor.................................ʒ j,

Acidi. hydrochlor., dil. Tr. ferri. chlor., } .................................aa ʒ ij,

Syr. Zingib.................................℥ ij,

Aquæ ad.................................℥ vj.

M. Sig. Two Teaspoonfuls three times daily in water, after meals.—*Canada Lancet.*

